# Improving Integrated Care: Can Implementation Science Unlock the ‘Black Box’ of Complexities?

**DOI:** 10.5334/ijic.4724

**Published:** 2019-07-25

**Authors:** Nick Goodwin

**Affiliations:** 1 International Foundation for Integrated Care, Wolfson College, Linton Road, Oxford, UK; 2 International Journal of Integrated Care, Wolfson College, Linton Road, Oxford, UK

**Keywords:** complexity, evaluation, methodology, implementation science, improvement, integrated care, social science

In a previous IJIC editorial I reflected on the fact that we have yet to make any significant breakthrough to understand the implementation and sustainability of complex service innovations that so characterise the development of integrated care programmes [1]. Without such knowledge we might be able to explain the core building blocks of integrated care systems, but we cannot adequately explain the intricacies of effective implementation nor fully understand the causes of the outcomes we observe. This is not simply a methodological problem but reflects a more deep-rooted challenge in the lack of value that is placed both in the commissioning of such research and the findings that are produced.

Last month I attended a one-day seminar in London, attended by a ‘who’s who’ of health service researchers, examining the *Challenges of Evaluating Integrated Care Programmes. This was* hosted by the Health Foundation and the Nuffield Trust and was a chance to reflect on the evidence for integrated care following the recent conclusion of the three-year New Care Models programme funded by NHS England [2,3,4]. This itself built on earlier and well evaluated programmes such as the Integrated Care Pilots [5] and Integrated Care Pioneers [6]. Specifically, the seminar asked why all this research had yet not been able to reach any definitive conclusion to how the impact (positive, negative or neutral) of different integrated care strategies could be explained?

For example, evidence was presented at the meeting that multi-speciality community providers (MCPs) – one of the new care models in England that sought to promote specialist care in the community – had had a collective impact in the lowering of emergency hospital admissions (if not overall bed days) [4]. However, given the significant variation in ‘the what and the how’ of implementation in practice, it has been impossible to really understand what interventions or processes have been able to make the difference, how these should be balanced in practice, or indeed at what cost [4]. This means we don’t really have enough knowledge on what or how to implement things, nor on what really makes a difference to the outcomes we observe – the very practicalities that managers and professionals need if they are to replicate or adopt new ways of working effectively.

The upshot of the discussion was that we needed research which allowed for a deeper dive into the ‘black box’ of complexities that integrated care initiatives present. However, it was also noted that evaluations, even those of the highest technical quality, rarely have been given the opportunity to use an implementation science approach to examine what happens at the operational level. Moreover, because such qualitative research is both time- and resource-intensive (for example, requiring ethnographic and observational methods) such investigations are often based on surveys and/or case study approaches of limited design. The consequence is that this results in a list of ‘truisms’ – e.g. ‘teamwork is essential’, ‘effective leadership matters’, or ‘success depended on a positive organisational culture’. Such findings give you an understanding of the outcome domains that matter when evaluating the difference between success and failure, but don’t solve the problem of the practical tools and approaches that might be used in specific contexts to build or replicate such capabilities (if required, of course, since some things happen naturally or even by fluke).

If we are serious about understanding the ‘black box’ of complexities, then we need to take social science seriously. Unfortunately, the value of an implementation science approach is under-appreciated, rarely commissioned and often not published in the ‘high impact’ journals as they are seen as lower on the evidence hierarchy from which decisions are made. This is despite the overwhelming evidence that it is the ‘softer’ issues such as leadership and management, effective teams and networks, and positive cultures and behaviours that are the most likely to be deciding factors in whether innovations in integrated care succeed or fail. Investigations, therefore, need to become much more granular in understanding how behaviours are influenced and the practical tools and approaches required to do so.

To take a step forward in the development of practical tools to support the implementation of integrated care in practice, a cross-European project called SUSTAIN (Sustainable Tailored Integrated Care for Older People in Europe) sought to understand what worked, and what didn’t work, when making improvements to highly variable integrated care initiatives across 7 different countries [7]. The results of the 4-year study found the usual suspects – for example, related to things like the impact of professional attitudes and relationships, the importance of well-functioning teams, cultural factors and local contexts.

However, as the SUSTAIN researchers conclude, *“the dynamic and non-linear nature of the improvement process must be recognised in order to take integrated care forward successfully. The environment within which integrated care programmes emerge in their own contexts change over time, and the agents (the people and organisations involved) are constantly adapting to such changes. Improvement processes help to reveal a range of issues that need to be addressed in order to implement services effectively and so achieve the improvement goals that integrated care initiatives seek to make. Whilst some of these issues may relate to minor changes in practice, others may reveal larger cultural or organisational issues that need to be addressed. An approach to integrated care improvement is therefore needed that is intelligent, sensitive, responsive and adaptive”* [8, p. 62].

Over the course of the project, SUSTAIN identified and piloted many approaches to improvement. The final SUSTAIN Roadmap helpfully provides an account of these improvement methods to provide a set of insights for others looking to implement similar approaches to improvement in their own contexts [8]. In this regard, key operational issues are addressed in detail – such as conducting meetings, establishing steering groups, enabling team momentum, communicating and measuring progress, and providing effective feedback. The research suggests that the engine room of successful integrated care programmes, as complex service innovations, requires a cycle of improvement since making progress is always non-linear and usually a messy affair.

Progress in implementation science methodology continues to be made that go beyond the variations to realistic synthesis reviewed previously [1], specifically in ways that help to recognise how and why the more intangible qualities of complex interventions might better explain outcomes. For example, the recently published NASSS framework has constructed a taxonomy for empirical application to help explain the non-adoption or abandonment of technology by individuals and difficulties achieving scale-up, scale and sustainability, an approach that has clear resonance for investigating integrated care [9]. Development evaluation is also a relatively new approach to support the creation of dynamic, complex innovations where expected outcomes are uncertain or unclear [10] and has been used quite successfully in the context of transforming care delivery in Foundry – a province-wide initiative in British Columbia, Canada, that involves over 100 partnerships target young people aged 12–24 to provide a one-stop-shop for integrated mental health care, substance use, primary care, social services, and youth and family peer support [11]. By creating a space for evaluation, the voices and priorities of young people in the co-design and development of the services could be created with decision-makers and funders over time [12].

The conclusion to be drawn is that programme evaluations, however well designed to work through economic and other impacts, are likely to have limited ability to explain how integrated care works in practice. Process evaluations within these will pick up on important key themes and issues in implementation, but not the tools and approaches that were used to enable them. What is needed is a shift in tactic where evaluation takes a more practical and participatory form to support continuous reflective learning that is embedded within integrated care projects and which act as a tool for quality improvement. Evaluation and monitoring practices may then become built-in to the DNA of everyday working practice, valued by all participants, and so enable the complexities of integrated care in specific contexts to be resolved in real-time.

Some might argue that this approach would be too expensive and time consuming or may lack objectivity and thus the ability to validate findings. Yet, as explained in this editorial, integrated care programmes are complex and dynamic entities that require reflexive enquiry in order to evolve and improve over time. The methodologies we use to assess them therefore need to capture these dynamics and promote a more intimate relationship between research and practice. This has some profound consequences since it implies that research needs to play the supportive (but not subordinate) role to practice, but equally that practice should respond to research findings in equal measure. Unlocking the ‘black box’ to improve our knowledge of implementation complexity therefore implies the need to simultaneously tackle the tensions inherent in action research [13].
